# Combined effects of an angiogenesis inhibitor (TNP-470) and hyperthermia.

**DOI:** 10.1038/bjc.1996.48

**Published:** 1996-02

**Authors:** Y. Nishimura, R. Murata, M. Hiraoka

**Affiliations:** Department of Radiology, Faculty of Medicine, Kyoto University, Japan.

## Abstract

**Images:**


British Journal of Cancer (1996) 73, 270-274
© 1996 Stockton Press All rights reserved 0007-0920/96 $12.00

### Combined effects of an angiogenesis inhibitor (TNP-470) and hyperthermia

Y Nishimura, R Murata and M Hiraoka

*Department of Radiology, Faculty of Medicine, Kyoto University, 54-Shogoin-Kawahara-cho, Sakyo-ku, Kyoto 606-01, Japan.*

**Summary** TNP-470, a synthetic analogue of fumagillin first isolated from *Aspergillus fumigatus*, is known to be a potent anti-angiogenic compound. The combined effects on tumour growth and tumour angiogenesis of TNP-470 and hyperthermia were investigated. The tumour used was SCCVII carcinoma of the C3H/He mouse. The tumour response was evaluated by the tumour growth (TG) time assay. The TG time is the time required for one-half of the treated tumours to reach three times the initial tumour volume. Significant delay of tumour growth was observed by TNP-470 alone (100 mg $kg^{-1} \times 2$ or $\times 4$), indicating that TNP-470 alone has anti-tumour effect *in vivo*. When TNP-470 (100 mg $kg^{-1} \times 2$ or $\times 4$) was administered after hyperthermia at 44°C, the TG times of the combined treatment were significantly longer than those of heat alone (44°C) or TNP-470 (100 mg $kg^{-1} \times 2$ or $\times 4$) alone. However, the TG time of combined treatment with TNP-470 and hyperthermia at 42°C was quite similar to that of TNP-470 alone. This conflicting result on the combined effect of TNP-470 and hyperthermia may be related to the temperature-dependent vascular damage by hyperthermia. Dose-dependent inhibition of angiogenesis by TNP-470 was demonstrated in microangiograms obtained 4 days and 7 days after hyperthermia (44°C for 30 min). It is, thus, suggested that the combined effect of TNP-470 and hyperthermia is attributable to the inhibition of angiogenesis by TNP-470 following heat-induced vascular damage.

**Keywords:** hyperthermia; heat-induced vascular damage; angiogenesis inhibitor; angiogenesis

Angiogenesis is critical for the growth of solid tumours and metastasis (Folkman, 1990). Therefore, anti-angiogenic therapy is probably one of the most promising strategies for restricting tumour growth (Denekamp, 1991; Bricknell and Harris, 1991; Brem and Folkman, 1993). TNP-470, a synthetic analogue of fumagillin first isolated from *Aspergillus fumigatus*, is known to be a potent anti-angiogenic compound (Ingber *et al.*, 1990; Kusaka *et al.*, 1991). It has been found to suppress not only endothelial cell growth *in vitro*, but also tumour growth *in vivo* (Brem and Folkman, 1993; Ingber *et al.*, 1990; Kusaka *et al.*, 1991, 1994; Toi *et al.*, 1994; Yanase *et al.*, 1993; Yamaoka *et al.*, 1993*a*,*b*). The anti-tumour effect *in vivo* was thought to be due to inhibition of angiogenesis.

Blood perfusion plays an important role in the tissue damage by hyperthermia. Tissue temperature is dependent on blood flow rate, and it also controls the intratumoral microenvironment, which affects the thermosensitivity of the tissue (Song, 1984; Reinhold and Endrich, 1986; Vaupel, 1988). In addition, hyperthermia itself selectively destroys the tumour vasculature at a high heat dose (Song, 1984; Reinhold and Endrich, 1986; Vaupel, 1988; Nishimura *et al.*, 1988*a*,*b*). In previous studies, we demonstrated that heating at 44°C for 30 min almost destroys murine tumour vasculature and angiogenesis occurs from the peripheral zone of the tumour 3 days after heating (Nishimura *et al.*, 1988*a*). Therefore, it is an interesting question whether inhibition of the angiogenesis by an angiogenesis inhibitor may enhance the effect of hyperthermia.

In the present study, we examined the effects of TNP-470 on hyperthermia-induced growth delay of SCCVII tumours. The effect of TNP-470 on tumour angiogenesis after hyperthermia was investigated quantitatively by using microangiography.

### Materials and methods

### *Animal tumour*

Eight-week old C3H/He male mice were used throughout the study. The mice were obtained from Shizuoka Laboratory Animal Center (Shizuoka, Japan), and kept in our micro-organism-free animal facility. They were provided with sterilised mouse pellets and water *ad libitum*.

SCCVII carcinoma of the C3H/He mice was used. The SCCVII tumour is a squamous carcinoma that arises spontaneously in the abdominal wall of a C3H mouse (Hirst *et al.*, 1982). The SCCVII tumour cell line was thawed from original frozen stocks and maintained by alternate passage in syngeneic mice and cell culture in Eagle's minimum essential medium supplemented with 12.5% fetal bovine serum. Some of the biological characteristics of the SCCVII tumour were described elsewhere (Hirst *et al.*, 1982). Approximately $1 \times 10^5$ SCCVII tumour cells collected from monolayer cultures were inoculated s.c. into the right thigh of the C3H/He male mice both for tumour growth (TG) time assay and for microangiography. Experiments were performed when the tumours reached 8-10 mm in diameter.

### *Drug*

TNP-470 was kindly provided by Takeda Chemical Industries, Osaka, Japan. TNP-470 was dissolved in 5% arabic gum-saline solution. For the TG time assay, two or four doses of TNP-470 (100 mg $kg^{-1}$) were administered s.c. into the back of the mice.When TNP-470 was administered in combination with hyperthermia, the first dose was given 3 h after heat treatment (day 0). Second, third and fourth doses were administered on days 3, 7 and 10 respectively. For analysis of tumour angiogenesis by microangiography, two doses of TNP-470 (50 mg $kg^{-1}$ or 100 mg $kg^{-1}$) were administered s.c. 3 h after hyperthermia (44°C for 30 min) and on day 3.

### *Hyperthermia*

Hyperthermia was achieved by immersing the animal's foot in a water bath. Intratumoral thermometry data have been described elsewhere (Nishimura *et al.*, 1988*a*). Generally, temperatures of thigh tumours were equilibrated within 3-4 min after immersion in the water bath, and remained 0.2-0.3°C below the water bath temperature. Mice were anaesthetised before hyperthermia by i.p. injection of pentobarbital (60 mg $kg^{-1}$), and the right tumour-bearing leg was pulled down by a sinker to the water bath. The mice were air-cooled during the heat treatment. All temperatures

Correspondence: Y Nishimura
Received 30 June 1995; revised 30 August 1995; accepted 14 September 1995

mentioned in this paper refer to the water bath temperature. In this study, two heat doses of 42°C for 30 min and 44°C for 30 min were used.

*Assays of tumour response*

The tumour response was evaluated by the tumour growth (TG) time assay. The TG time assay measures the time required for a tumour to reach 3-fold initial tumour volume from the first day of treatment. Three diameters of a tumour, *a*, *b* and *c* were measured using a calliper every 2 days after various treatments, and the volume was calculated by a formula; $\pi abc/6$. Tumour volume was measured until the tumour reached 2000 $mm^3$. The TG time was determined for individual tumours, and the median TG time with 95% confidence limits (CLs) was calculated for each treatment group by logit analysis based on the relationship between the cumulative percentage of tumours that reached 3-fold initial tumour volume and the days after treatment (Nishimura and Urano, 1993). Each treatment group consisted of 10–12 mice. Statistical analysis between the treatment groups was performed using Student's *t*-test, and $P<0.05$ was regarded as significant difference.

*Analysis of tumour angiogenesis*

Vascular damage and angiogenesis after hyperthermia were evaluated by microangiography and correlative histological sections. The technique of microangiography was described previously (Nishimura *et al*., 1988*a*). Briefly, a filtered barium sulphate solution (0.25 g $ml^{-1}$) was injected at a pressure of 150 mmHg after flushing the circulatory system with warmed heparinised saline. When the muscular vasculature of the hind limb was not opacified sufficiently, the mouse was excluded from the study to avoid a poor filling artifact. In the present study, 2 of 42 mice used for microangiography were excluded from the analysis because of the poor filling artifact. After the tumour was fixed with 10% buffered formalin, contact radiographs of 1-mm-thick tumour slices were obtained. The tumour slice was cut along a sagittal direction through its centre with as much surrounding normal tissue as posssible. Correlative histological sections 4 $\mu m$ thick were prepared for each tumour slice and stained with haematoxylin and eosin.

Microangiographic changes were analysed quantitatively as follows (Nishimura *et al*., 1988*a*,*b*). Opacified vascularised areas and avascular areas in a tumour were demarcated on an enlarged microangiogram (approximately × 10), and the vascularised areas (V) and the entire tumour area (T) were measured using a digital planimeter. Thereafter, the percentage of vascularised area was calculated as V/T × 100 in each microangiogram. The mean and s.e. of the percentage of vascularised area was obtained for each group. Each group consisted of 4–9 angiograms (mean 6).

### Results

*TG time assay*

The results of TG time assay for TNP-470 are shown in Table I. The TG times of control tumours and those treated with TNP-470 (100 mg $kg^{-1}$ × 2) were 7.3 days (95% CL: 6.6–8.0 days) and 8.7 days (8.3–9.2 days) respectively. The difference between these two TG times was statistically significant ($P<0.05$), indicating that TNP-470 alone has an anti-tumour effect *in vivo*. When four doses of TNP-470 were administered, the TG time was further increased.

Figures 1 and 2 show the tumour growth curves for tumours treated with hyperthermia at 42°C and 44°C with or without TNP-470 (100 mg $kg^{-1}$ × 2). Hyperthermia alone at 42°C did not result in any growth delay compared with control tumours, while hyperthermia at 44°C resulted in significant growth delay compared with untreated control tumours ($P<0.05$). When TNP-470 was administered following hyperthermia at 42°C, the tumour growth curve was not different from the growth curve for tumours treated with TNP-470 alone (Figure 1). However, when TNP-470 was administered after hyperthermia at 44°C, significant tumour growth delay was observed compared with those for each treatment alone (Figure 2). The TG time of heat at 44°C and TNP-470 (100 mg $kg^{-1}$ × 2) was significantly longer than those of heat alone at 44°C or TNP-470 (100 mg $kg^{-1}$ × 2) alone ($P<0.01$). Interestingly, tumour growth curves of hyperthermia combined with TNP-470 became parallel to

**Table I** TG time assay of TNP-470 and hyperthermia, SCCVII tumour

| *Treatment* | n | *TG time (days)* | *(95% CL)* |
|---|---|---|---|
| Control | 10 | 7.3 | (6.6–8.0) |
| TNP-470 (100 mg $kg^{-1}$ × 2) | 10 | 8.7 | (8.3–9.2) |
| TNP-470 (100 mg $kg^{-1}$ × 4) | 10 | 11.8 | (10.8–12.9) |
| HT alone (42°C) | 10 | 6.6 | (6.1–7.1) |
| HT (42°C) + TNP-470 (100 mg $kg^{-1}$ × 2) | 11 | 8.9 | (8.4–9.5) |
| HT alone (44°C) | 11 | 8.6 | (8.0–9.3) |
| HT (44°C) + TNP-470 (100 mg $kg^{-1}$ × 2) | 11 | 10.4 | (9.9–10.8) |
| HT (44°C) + TNP-470 (100 mg $kg^{-1}$ × 4) | 11 | 16.4 | (14.9–18.1) |

HT, hyperthermia, heating time was 30 min; *n* = number of mice.

**Figure 1** Tumour growth curves for SCCVII tumours treated with hyperthermia (42°C for 30 min) with or without TNP-470 (100 mg $kg^{-1}$ × 2); control tumours (○), hyperthermia alone (□), TNP-470 alone (●), hyperthermia plus TNP-470 (■). Hyperthermia was applied on day 0, and arrows indicate the date of administration of TNP-470. Vertical bars = s.e.

**Figure 2** Tumour growth curves for SCCVII tumours treated with hyperthermia (44°C for 30 min) with or without TNP-470 (100 mg $kg^{-1}$ × 2); control tumours (○), hyperthermia alone (□), TNP-470 alone (●), hyperthermia plus TNP-470 (■). Hyperthermia was applied on day 0, and arrows indicate the date of administration of TNP-470. Vertical bars = s.e.

that of control tumours several days after the last dose of TNP-470, indicating that the growth delay was closely related to the administration of TNP-470.

Figure 3 shows the tumour growth curves for tumours treated with hyperthermia at 44°C with or without TNP-470 (100 mg $kg^{-1} \times 4$). Although administration of four doses of TNP-470 caused apparent suppression of the tumour growth compared with untreated control tumours, tumour growth was not inhibited completely. On the other hand, when TNP-470 was administered following hyperthermia at 44°C, complete inhibition of tumour growth was observed for 6 days after hyperthermia. The median TG time of the combination was 16.4 days (14.9–18.1 days), which was significantly longer than those of TNP-470 alone (100 mg $kg^{-1} \times 4$) or hyperthermia alone at 44°C ($P < 0.001$).

*Tumour angiogenesis*

Figure 4 shows the changes in percentage of vascularised area after heating at 44°C for 30 min. In the previous study, percentage of vascularised area of untreated SCCVII carcinoma was $100 \pm 0\%$, and no focal avascular area or necrotic area was observed histologically for untreated SCCVII tumours (Nishimura *et al.*, 1988*a*). The percentage of vascularised area decreased to $6 \pm 2\%$ at 1 day after heating, indicating that tumour vasculature of SCCVII carcinoma was destroyed nearly completely by the heat dose (Figure 5a). The percentage of vascularised area increased rapidly at day 4 and day 7 for tumours treated with hyperthermia alone. In the microangiograms, tapering capillary sprouts indicating angiogenesis were observed at the inner edge of the opacified vascularised area (Figure 5b).

For tumours treated with TNP-470 (50 mg $kg^{-1}$ or 100 mg $kg^{-1}$) following hyperthermia, the percentage of vascularised area increased more slowly than tumours treated with hyperthermia alone (Figures 4 and 5). Although complete inhibition of angiogenesis was not observed even when 100 mg $kg^{-1}$ TNP-470 was administered twice weekly, the percentage of vascularised area of tumours treated with TNP-470 (100 mg $kg^{-1}$) at day 4 and day 7 were significantly smaller than those of tumours treated with hyperthermia alone at day 4 and day 7 ($P = 0.005$ and $P = 0.018$ respectively). The difference in the percentage of vascularised area at day 4 was borderline significance between TNP-470 (50 mg $kg^{-1}$) and the hyperthermia alone group ($P = 0.08$). Thus, the rate of tumour angiogenesis was inhibited by TNP-470 in a dose-dependent manner.

**Figure 3** Tumour growth curves for SCCVII tumours treated with hyperthermia (44°C for 30 min) with or without TNP-470 (100 mg $kg^{-1} \times 4$); control tumours (○), hyperthermia alone (□), TNP-470 alone (●), hyperthermia plus TNP-470 (■). Hyperthermia was applied on day 0, and arrows indicate the date of administration of TNP-470. Vertical bars = s.e.

**Figure 4** Changes in percentage of vascularised area of SCCVII tumours following heating at 44°C for 30 min with or without TNP-470: no TNP-470 (■), TNP-470 (50 mg $kg^{-1} \times 2$) (△), TNP-470 (100 mg $kg^{-1} \times 2$) (●). Hyperthermia was applied on day 0, and arrows indicate the date of administration of TNP-470. Vertical bars = s.e.

### Discussion

Anti-angiogenic therapy by an angiogenesis inhibitor, TNP-470, has been studied extensively since Ingber *et al.* (1990) demonstrated that TNP-470 exhibited inhibitory activity on endothelial cells and solid tumour growth (Brem and Folkman, 1993; Kusaka *et al.*, 1991, 1994; Toi *et al.*, 1994; Yanase *et al.*, 1993; Yamaoka *et al.*, 1993*a*,*b*). TNP-470 is one of the synthetic analogues of fumagillin and shows more potent anti-angiogenic activity and less toxicity than fumagillin (Kusaka *et al.*, 1991). Kusaka *et al.* (1994) demonstrated that TNP-470 inhibited the growth of endothelial cells in a biphasic manner. The inhibition was cytostatic in the first phase at a concentration range of 0.3–3000 ng $ml^{-1}$, and it was cytotoxic in the second phase (> 30 $\mu g$ $ml^{-1}$). Because the serum concentration of TNP-470 *in vivo* was much lower than that for the cytotoxic inhibition (Kusaka *et al.*, 1994), TNP-470 should be combined with other cytotoxic anti-cancer agents in cancer therapy. Combined effects of TNP-470 and chemotherapy or hormone therapy have been demonstrated in several *in vivo* studies so far (Yamaoka *et al.*, 1993*a*,*b*; Teicher *et al.*, 1994; Kamei *et al.*, 1993; Toi *et al.*, 1993). In this study, the combined effects of TNP-470 and hyperthermia were examined.

Significant growth delay was observed in the TG time assay by administrating TNP-470 (100 mg $kg^{-1}$) after hyperthermia at 44°C compared with either TNP-470 alone or hyperthermia alone (Figures 2 and 3). Blood flow plays the most important role in the hyperthermic damage in tissues, including tumours. An important fact is that heat causes profound changes in tumour vasculature and blood flow (Song, 1984; Reinhold and Endrich, 1986; Vaupel *et al*, 1988; Nishimura *et al.*, 1988*a*,*b*). A number of studies demonstrated that vascular beds in experimental rodent tumours as well as those in human tumour xenografts are more vulnerable to heat compared with those in normal tissues (Song, 1984; Reinhold and Endrich, 1986). In the previous study, we demonstrated that heating at 44°C for 30 min destroys tumour vasculature of the SCCVII tumour nearly completely and tumour angiogenesis occurs from the peripheral zone of the tumour 3 days after heating (Nishimura *et al.*, 1988*a*). On the other hand, muscle vasculature was well opacified either after hyperthermia alone or hyperthermia combined with TNP-470 (Figure 5). As TNP-470 has anti-angiogenic activity against rapidly proliferating endothelium, including tumour neovascularisation, this drug may have little effect on physiological states of normal vasculatures except for ovulation, menstruation and the development of the placenta (Folkman, 1985). Thus, it is very likely that TNP-470 prolonged the growth delay by inhibiting the tumour angiogenesis following hyperthermia.

As shown in Figures 2 and 3, growth rate of tumours

**Figure 5** Microangiograms of SCCVII tumours. (**a**) One day after hyperthermia (44°C for 30 min). Tumour vessels showed nearly no filling, although muscular vessels were well opacified. (**b**) Seven days after hyperthermia alone. A small avascular area was surrounded by extensive angiogenesis. Tapering capillary sprouts were well observed at the inner edge of the opacified vascularised area. (**c**) Seven days after hyperthermia treated with TNP-470 (50 $mg kg^{-1} \times 2$). Although angiogenesis was also noted, a moderate avascular area was present. (**d**) Seven days after hyperthermia treated with TNP-470 (100 $mg kg^{-1} \times 2$). A wide avascular area was still present, indicating inhibition of angiogenesis by TNP-470.

treated with hyperthermia and TNP-470 returned to the same level as that of control tumours several days after the last dose of TNP-470. This indicates that the inhibition of angiogenesis by TNP-470 was cytostatic and endothelial cells regrew after the cessation of administration of TNP-470. Such cytostatic inhibition of endothelial cells by TNP-470 and regrowth after removal of the drug was well demonstrated *in vitro* (Kusaka *et al.*, 1994).

Although the combined effect of TNP-470 and hyperthermia was noted at 44°C heating, it was not demonstrated at 42°C (Figures 1 and 2). Apparently vascular damage by hyperthermia is temperature dependent (Song, 1984; Reinhold and Endrich, 1986; Vaupel, 1988; Nishimura *et al.*, 1988*a*). In the previous study, we demonstrated that 95% of tumour vasculature of the SCCVII tumour was destroyed 24 h after heating at 44°C, whereas only 24% of tumour vasculature was destroyed 24 h after heating at 42°C (Nishimura *et al.*, 1988*a*). Because heat effects are also time dependent, severe vascular damage may occur when hyperthermia is applied for longer periods at 42°C. It is apparent that heat dose is more important than the heating temperature itself. TNP-470 might have greater effects after a high heat dose than a low heat dose, because significant portions of tumour vasculature should be damaged by hyperthermia before administrating an angiogenesis inhibitor.

In clinical hyperthermia only limited portions of the tumour volume can be heated to 43–44°C even for superficial tumours. In addition, the tumour vasculature of human tumours is considered to be more resistant to heat than the murine tumour vasculature (Waterman *et al.*, 1987). Thus, it remains unclear whether this particular combination between TNP-470 and high temperature hyperthermia will have clinical relevance. However, CT scans obtained after clinical thermoradiotherapy showed a clear low-density area in well-heated human tumours, which was confirmed histologically to be coagulation necrosis (Hiraoka *et al.*, 1987; Nishimura *et al.*, 1989). Leopold *et al.* (1992) reported that 60% of human soft tissue sarcomas treated with preoperative thermoradiotherapy showed tumour necrosis of >80% area in resected specimens. Jo *et al.* (1989) demonstrated that small tumour blood vessels and capillaries in human tumour parenchyma were markedly damaged after thermoradiotherapy. Thus, it is likely that human tumour vasculatures may be severely damaged by several sessions of satisfactory heating combined with radiation therapy. Therefore, TNP-470 may be clinically useful if it is combined with thermoradiotherapy of good heating quality.

In the analysis of tumour angiogenesis by microangiography, significant inhibition of angiogenesis was observed by TNP-470. Although inhibition of angiogenesis by TNP-470 has been demonstrated by a vascular grading system in human nerve sheath tumours (Takamiya *et al.*, 1993), this study first showed that TNP-470 inhibited tumour angiogenesis *in vivo* quantitatively. This analysis system of tumour angiogenesis after hyperthermia seems a good model to study angiogenesis of murine tumour vasculature *in vivo* because hyperthermia at a high heat dose inevitably destroys murine tumour vasculature followed by angiogenesis. The effect of TNP-470 alone on spontaneously growing tumour vasculature was not studied in the microangiography because rapid tumour angiogenesis was not observed in microangiograms without initial vascular damage by hyperthermia.

Dose-dependent inhibition of angiogenesis by TNP-470 was clearly demonstrated in Figure 4. However, complete inhibition of angiogenesis was not observed even when 100 mg $kg^{-1}$ TNP-470 was administered twice weekly. Because complete inhibition of endothelial cell growth by TNP-470 was noted as low as 0.3 ng $ml^{-1}$ *in vitro* (Kusaka *et al.*, 1994; Toi *et al.*, 1994), the concentration of TNP-470 at the tumour tissue may be less than that level. On the other hand, complete inhibition of tumour growth by TNP-470 was observed for 6 days after hyperthermia at 44°C in the TG time assay (Figure 3), although tumour regrowth started when third and fourth doses of TNP-470 were still administered. As shown in Figures 4 and 5, tumour angiogenesis was progressing slowly by replacing necrotic area with viable tumour cells even when the tumour volume was not changed. Thus, changes in tumour volume began to occur after a substantial portion of necrotic area was replaced by viable tumour cells. Therefore, it is unlikely that the tumour endothelial cells become resistant to TNP-470 after administration of several doses. To obtain complete inhibition of tumour angiogenesis, administration of TNP-470 with shorter interval and/or higher doses may be necessary.

In the present study, TNP-470 was given after hyperthermia to investigate its effects on tumour angiogenesis. However, TNP-470 may enhance heat effects when it is administered before heating. Inhibition of tumour angiogenesis by TNP-470 may make the intratumoral environment more acidic and hypoxic, which increases the hyperthermic damage to tissues (Song, 1984; Reinhold and Endrich, 1986;Vaupel, 1988). Further study to confirm this hypothesis is in progress.

In conclusion, our results indicated that the greater tumour growth delay produced by the combination of TNP-470 and hyperthermia at 44°C resulted from the inhibition of tumour angiogenesis following heat-induced vascular damage. As hyperthermia is a clinically applicable vascular-damaging agent, the combination of hyperthermia and anti-angiogenic treatment seems a promising strategy.

**Acknowledgements**
This work was supported in part by Grant-in-Aid for Scientific Research (05151039) from the Ministry of Education, Science and Culture, Japan.

### References

BREM H AND FOLKMAN J. (1993). Analysis of experimental antiangiogenic therapy. *J. Pediatric. Surg.*, **28,** 445–451.

BRICKNELL R AND HARRIS AL. (1991). Novel growth regulatory factors and tumour angiogenesis. *Eur. J. Cancer*, **27,** 781–785.

DENEKAMP J. (1991). The current status of targeting tumour vasculature as a means of cancer therapy: an overview. *Int. J. Radiat. Biol.*, **60,** 401–408.

FOLKMAN J. (1985). Tumor angiogenesis. *Adv. Cancer Res.*, **43,** 175–203.

FOLKMAN J. (1990). What is the evidence that tumors are angiogenesis dependent? *J. Natl Cancer Inst.*, **82,** 4–6.

HIRAOKA M, AKUTA K, NISHIMURA Y, NAGATA Y, JO S, TAKAHASHI M AND ABE M. (1987). Tumor response to thermoradiation therapy: use of CT in evaluation. *Radiology*, **164,** 259–262.

HIRST DG, BROWN JM AND HAZLEHURST JL. (1982). Enhancement of CCNU cytotoxicity by misonidazole: possible therapeutic gain. *Br. J. Cancer*, **46,** 109–116.

INGBER D, FUJITA T, KISHIMOTO S, SUDO K, KANAMARU T, BREM H AND FOLKMAN J. (1990). Synthetic analogues of fumagillin that inhibit angiogenesis and suppress tumor growth, *Nature*, **348,** 555–557.

JO S, HIRAOKA M, AKUTA K, NISHIMURA Y, TAKAHASHI M, NISHIDA H, FURUTA M AND ABE M. (1989). Histopathological changes of human tumors following thermoradiotherapy. *Int.J. Radiat. Oncol. Biol. Phys.*, **17,** 1265–1271.

KAMEI S, OKADA H, INOUE Y, YOSHIOKA T, OGAWA Y AND TOGUCHI H. (1993). Antitumor effects of angiogenesis inhibitor TNP-470 in rabbits bearing VX-2 carcinoma by arterial administration of microspheres and oil solution. *J. Pharmacol. Exp. Ther.*, **264,** 469–474.

KUSAKA M, SUDO K, FUJITA T, MARUI S, ITOH F, INGBER D AND FOLKMAN J. (1991). Potent anti-angiogenic action of AGM-1470: comparison to the fumagillin parent. *Biochem. Biophys. Res. Commun.*, **174,** 1070–1076.

KUSAKA M, SUDO K, MATSUTANI E, KOZAI Y, MARUI S, FUJITA T, INGBER D AND FOLKMAN J. (1994). Cytostatic inhibition of endothelial cell growth by the angiogenesis inhibitor TNP-470 (AGM-1470). *Br. J. Cancer*, **69,** 212–216.

LEOPOLD KA, DEWHIRST M, SAMULSKI T, HARRELSON J, TUCKER JA, GEORGE SL, DODGE RK, GRANT W, CLEGG S, PROSNITZ LR AND OLESON JR. (1992). Relationships among tumor temperature, treatment time, and histopathological outcome using preoperative hyperthermia with radiation in soft tissue sarcomas. *Int. J. Radiat. Oncol. Biol. Phys.*, **22,** 989–998.

NISHIMURA Y, HIRAOKA M, JO S, AKUTA K, YUKAWA Y, SHIBAMOTO Y, TAKAHASHI M AND ABE M. (1988*a*). Microangiographic and histologic analysis of the effects of hyperthermia on murine tumor vasculature. *Int. J. Radiat. Oncol. Biol. Phys.*, **15,** 411–420.

NISHIMURA Y, SHIBAMOTO Y, JO S, AKUTA K, HIRAOKA M, TAKAHASHI M AND ABE M. (1988*b*). Relationship between heat-induced vascular damage and thermosensitivity in four mouse tumors. *Cancer Res.*, **48,** 7226–7230.

NISHIMURA Y, HIRAOKA M, JO S, AKUTA K, NAGATA Y, MASUNAGA S, TAKAHASHI M AND ABE M. (1989). Radiofrequency (RF) capacitive hyperthermia combined with radiotherapy in the treatment of abdominal and pelvic deep-seated tumors. *Radiother. Oncol.*, **16,** 139–149.

NISHIMURA Y AND URANO M. (1993). Timing and sequence of hyperthermia in fractionated radiotherapy of a murine fibrosarcoma. *Int. J. Radiat. Oncol. Biol. Phys.*, **27,** 605–611.

REINHOLD HS AND ENDRICH B. (1986). Tumor microcirculation as a target for hyperthermia. *Int. J. Hyperthermia*, **2,** 111–137.

SONG CW. (1984). Effect of local hyperthermia on blood flow and microenvironment: A review. *Cancer Res.*, **44** (suppl.), 4721s–4730s.

TAKAMIYA Y, FRIEDLANDER RM, BREM H, MALICK A AND MARTUZA RL. (1993). Inhibition of angiogenesis and growth of human nerve-sheath tumors by AGM-1470. *J. Neurosurg.*, **78,** 470–476.

TEICHER BA, HOLDEN SA, ARA G, SOTOMAYOR EA, HUANG ZD, CHEN YN AND BREM H. (1994). Potentiation of cytotoxic cancer therapies by TNP-470 alone and with other anti-angiogenic agents. *Int. J. Cancer*, **57,** 1–6.

TOI M, YAMAOKA Y, IMAZAWA T, TAKAYANAGI T, AKUTSU K AND TOMINAGA T. (1993). Antitumor effect of the angiogenesis inhibitor AGM-1470 and its combination effect with tamoxifen in DMBA induced mammary tumors in rats. *Int. J. Oncol.*, **3,** 525–528.

TOI M, TAKAYANAGI T, SOUMA R AND TOMINAGA T. (1994). Inhibition of vascular endothelial growth factor induced cell growth by an angiogenesis inhibitor AGM-1470 in capillary endothelial cells. *Oncol. Rep.*, **1,** 423–426.

VAUPEL P, KALLINOWSKI F AND KLUGE M. (1988). Pathophysiology of tumors in hyperthermia. *Recent Results Cancer Res.*, **107,** 65–75.

WATERMAN FM, NERLINGER RE, MOYLAN III DJ AND LEEPER DB. (1987). Response of human tumor blood flow to local hyperthermia. *Int. J. Radiat. Oncol. Biol. Phys.*, **13,** 75–82.

YANASE T, MASAKI T, FUJITA K, KODAMA S AND TANAKA K. (1993). Inhibitory effect of angiogenesis inhibitor TNP-470 on tumor growth and metastasis of human cell lines in vitro and in vivo. *Cancer Res.*, **53,** 2566–2570.

YAMAOKA M, YAMAMOTO T, MASAKI T, IKEYAMA S, SUDO K AND FUJITA T. (1993*a*). Inhibition of tumor growth and metastasis of rodent tumors by the angiogenesis inhibitor O-(chloroacetyl-carbamoyl) fumagillol (TNP-470; AGM-1470). *Cancer Res.*, **53,** 4262–4267.

YAMAOKA M, YAMAMOTO T, IKEYAMA S, SUDO K AND FUJITA T. (1993*b*). Angiogenesis inhibitor TNP-470 (AGM-1470) potently inhibits the tumor growth of hormone-independent human breast and prostate carcinoma cell lines. *Cancer Res.*, **53,** 5233–5236.


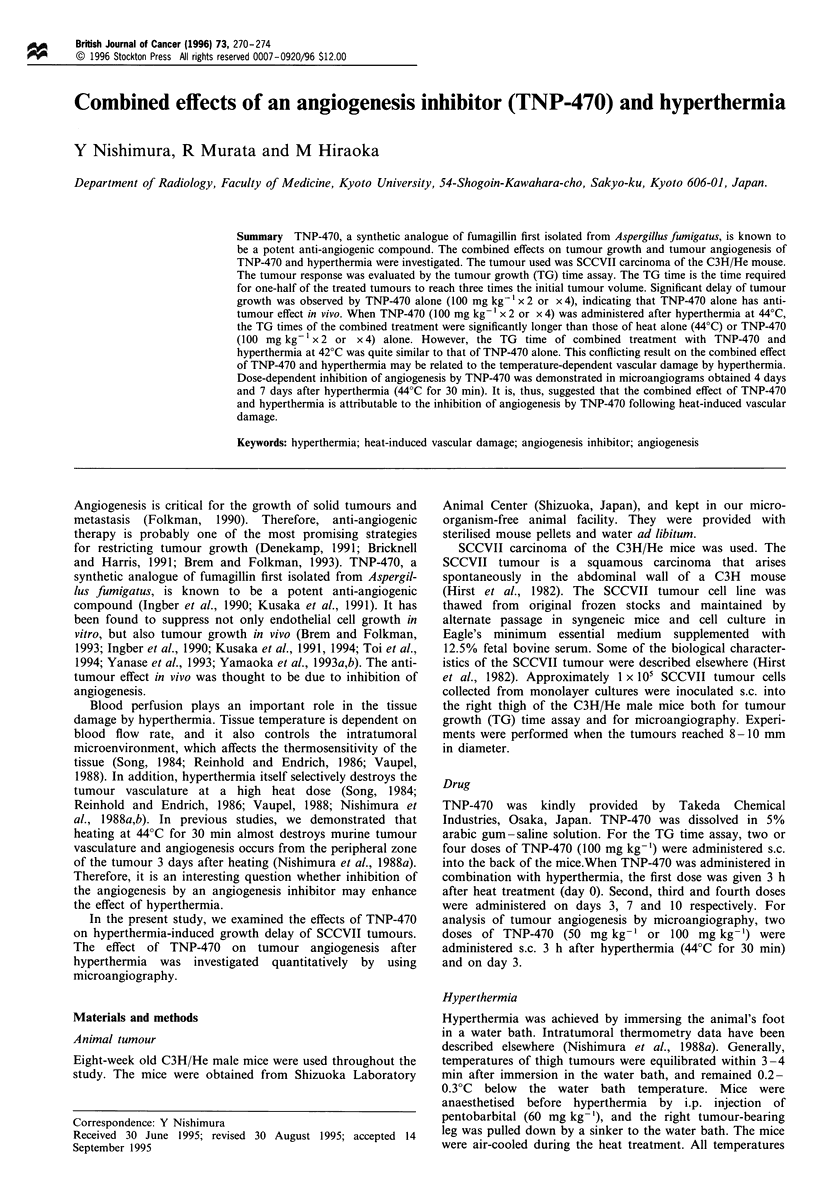

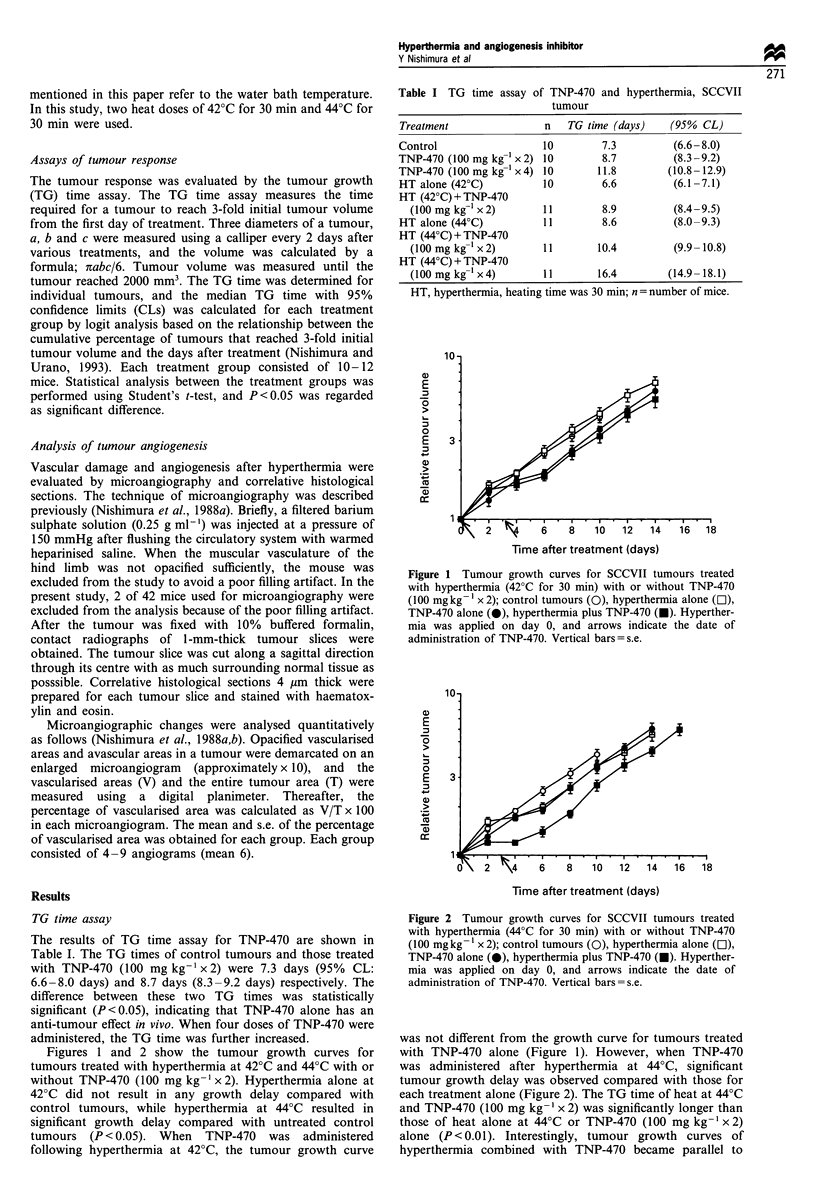

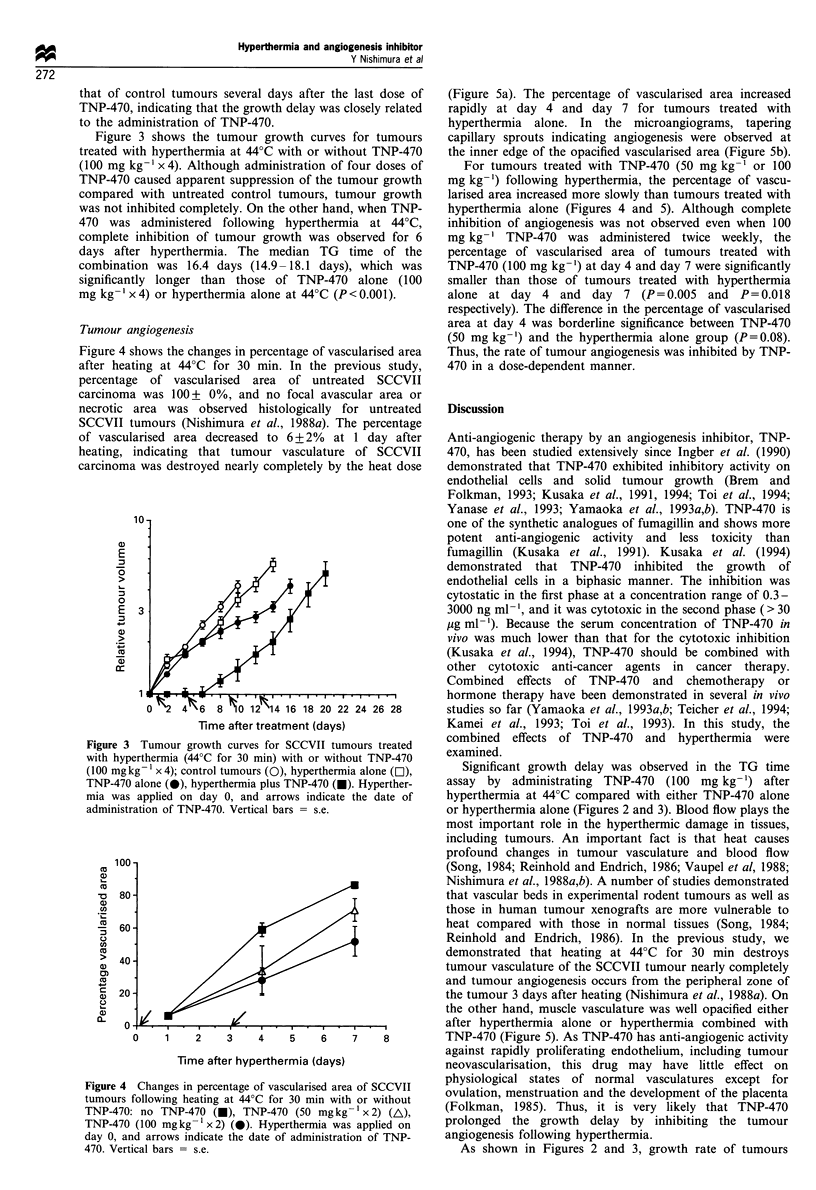

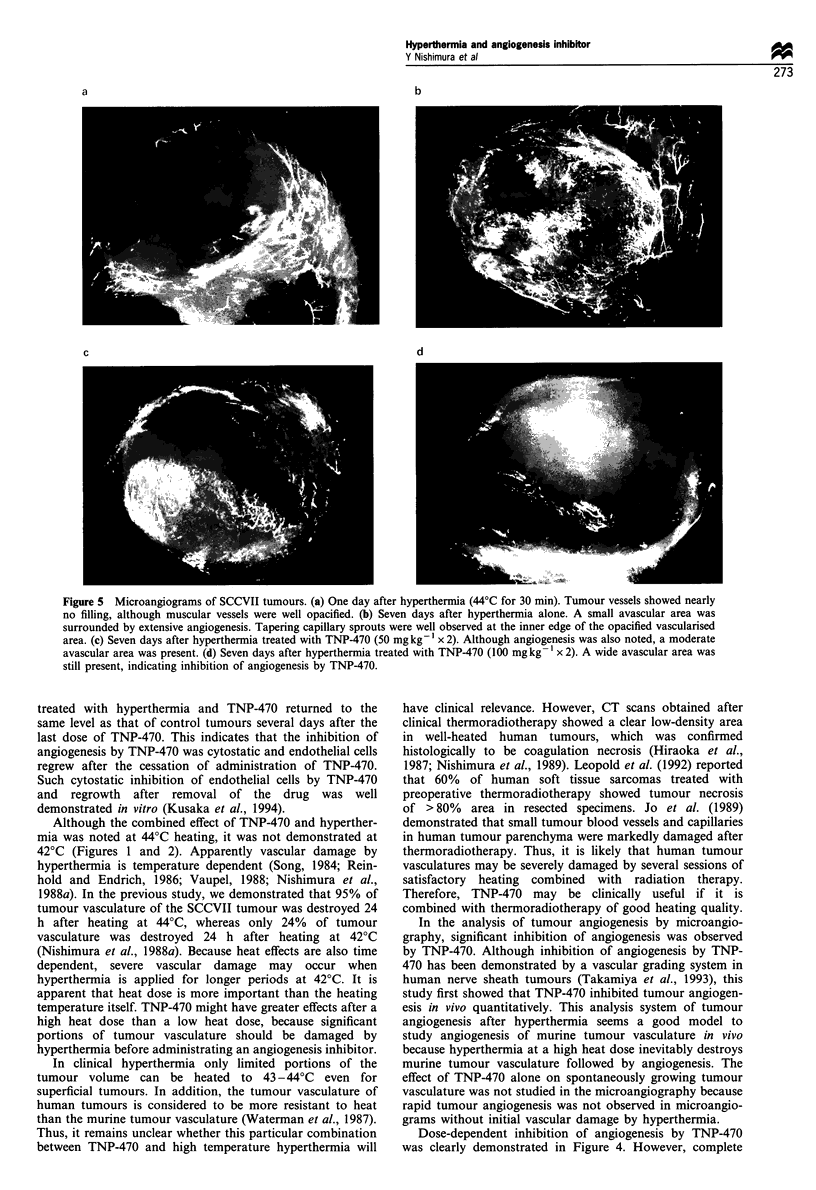

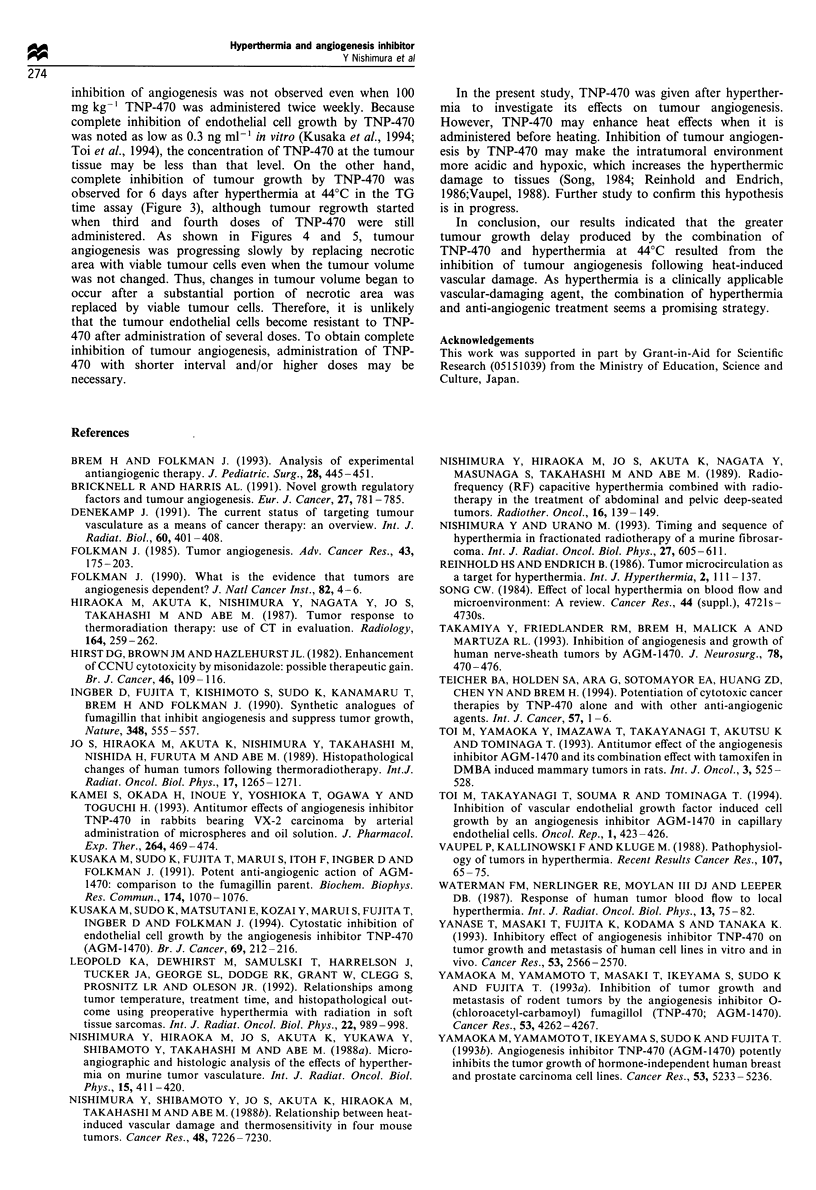


## References

[OCR_00632] Bicknell R., Harris A. L. (1991). Novel growth regulatory factors and tumour angiogenesis.. Eur J Cancer.

[OCR_00628] Brem H., Folkman J. (1993). Analysis of experimental antiangiogenic therapy.. J Pediatr Surg.

[OCR_00636] Denekamp J. (1991). The current status of targeting tumour vasculature as a means of cancer therapy: an overview.. Int J Radiat Biol.

[OCR_00639] Folkman J. (1985). Tumor angiogenesis.. Adv Cancer Res.

[OCR_00650] Hiraoka M., Akuta K., Nishimura Y., Nagata Y., Jo S., Takahashi M., Abe M. (1987). Tumor response to thermoradiation therapy: use of CT in evaluation.. Radiology.

[OCR_00655] Hirst D. G., Brown J. M., Hazlehurst J. L. (1982). Enhancement of CCNU cytotoxicity by misonidazole: possible therapeutic gain.. Br J Cancer.

[OCR_00661] Ingber D., Fujita T., Kishimoto S., Sudo K., Kanamaru T., Brem H., Folkman J. (1990). Synthetic analogues of fumagillin that inhibit angiogenesis and suppress tumour growth.. Nature.

[OCR_00667] Jo S., Hiraoka M., Akuta K., Nishimura Y., Takahashi M., Nishida H., Furuta M., Abe M. (1989). Histopathological changes of human tumors following thermoradiotherapy.. Int J Radiat Oncol Biol Phys.

[OCR_00673] Kamei S., Okada H., Inoue Y., Yoshioka T., Ogawa Y., Toguchi H. (1993). Antitumor effects of angiogenesis inhibitor TNP-470 in rabbits bearing VX-2 carcinoma by arterial administration of microspheres and oil solution.. J Pharmacol Exp Ther.

[OCR_00680] Kusaka M., Sudo K., Fujita T., Marui S., Itoh F., Ingber D., Folkman J. (1991). Potent anti-angiogenic action of AGM-1470: comparison to the fumagillin parent.. Biochem Biophys Res Commun.

[OCR_00683] Kusaka M., Sudo K., Matsutani E., Kozai Y., Marui S., Fujita T., Ingber D., Folkman J. (1994). Cytostatic inhibition of endothelial cell growth by the angiogenesis inhibitor TNP-470 (AGM-1470).. Br J Cancer.

[OCR_00693] Leopold K. A., Dewhirst M., Samulski T., Harrelson J., Tucker J. A., George S. L., Dodge R. K., Grant W., Clegg S., Prosnitz L. R. (1992). Relationships among tumor temperature, treatment time, and histopathological outcome using preoperative hyperthermia with radiation in soft tissue sarcomas.. Int J Radiat Oncol Biol Phys.

[OCR_00710] Nishimura Y., Hiraoka M., Jo S., Akuta K., Nagata Y., Masunaga S., Takahashi M., Abe M. (1989). Radiofrequency (RF) capacitive hyperthermia combined with radiotherapy in the treatment of abdominal and pelvic deep-seated tumors.. Radiother Oncol.

[OCR_00700] Nishimura Y., Hiraoka M., Jo S., Akuta K., Yukawa Y., Shibamoto Y., Takahashi M., Abe M. (1988). Microangiographic and histologic analysis of the effects of hyperthermia on murine tumor vasculature.. Int J Radiat Oncol Biol Phys.

[OCR_00706] Nishimura Y., Shibamoto Y., Jo S., Akuta K., Hiraoka M., Takahashi M., Abe M. (1988). Relationship between heat-induced vascular damage and thermosensitivity in four mouse tumors.. Cancer Res.

[OCR_00717] Nishimura Y., Urano M. (1993). Timing and sequence of hyperthermia in fractionated radiotherapy of a murine fibrosarcoma.. Int J Radiat Oncol Biol Phys.

[OCR_00724] Reinhold H. S., Endrich B. (1986). Tumour microcirculation as a target for hyperthermia.. Int J Hyperthermia.

[OCR_00728] Song C. W. (1984). Effect of local hyperthermia on blood flow and microenvironment: a review.. Cancer Res.

[OCR_00734] Takamiya Y., Friedlander R. M., Brem H., Malick A., Martuza R. L. (1993). Inhibition of angiogenesis and growth of human nerve-sheath tumors by AGM-1470.. J Neurosurg.

[OCR_00756] Vaupel P., Kallinowski F., Kluge M. (1988). Pathophysiology of tumors in hyperthermia.. Recent Results Cancer Res.

[OCR_00763] Waterman F. M., Nerlinger R. E., Moylan D. J., Leeper D. B. (1987). Response of human tumor blood flow to local hyperthermia.. Int J Radiat Oncol Biol Phys.

[OCR_00779] Yamaoka M., Yamamoto T., Ikeyama S., Sudo K., Fujita T. (1993). Angiogenesis inhibitor TNP-470 (AGM-1470) potently inhibits the tumor growth of hormone-independent human breast and prostate carcinoma cell lines.. Cancer Res.

[OCR_00772] Yamaoka M., Yamamoto T., Masaki T., Ikeyama S., Sudo K., Fujita T. (1993). Inhibition of tumor growth and metastasis of rodent tumors by the angiogenesis inhibitor O-(chloroacetyl-carbamoyl)fumagillol (TNP-470; AGM-1470).. Cancer Res.

[OCR_00768] Yanase T., Tamura M., Fujita K., Kodama S., Tanaka K. (1993). Inhibitory effect of angiogenesis inhibitor TNP-470 on tumor growth and metastasis of human cell lines in vitro and in vivo.. Cancer Res.

